# Implementation and evaluation of a family planning intervention engaging mothers-in-law of young women in India: a mixed methods pilot study

**DOI:** 10.1080/16549716.2025.2554435

**Published:** 2025-09-08

**Authors:** Lakshmi Gopalakrishnan, Usha Choudhary, Janelli Vallin, Debangana Das, Payal Sharma, Ankur Kachhwaha, Tamera Panjalingam, Richa Ruwala, Nadia Diamond-Smith

**Affiliations:** a Institute for Global Health Sciences, University of California, San Francisco, CA, USA; b Vikalp Sansthan, Udaipur, India; c Neerman, Mumbai, India; d Orange Tree Foundation, Jodhpur, India; e School of Public Health, University of California, Berkeley, CA, USA

**Keywords:** newly married women, daughters-in-law, South Asia, intergenerational, multigenerational intervention, gender transformative program, life skills and health empowerment, gender norms, gender equitable attitudes

## Abstract

**Background:**

Despite evidence that mothers-in-law (MILs) influence daughters-in-law’s (DILs) fertility and family planning decisions in South Asia, emphasizing early fertility and male grandchildren, few reproductive health interventions engage MILs directly.

**Objectives:**

We assessed the feasibility, acceptability, and qualitative impact of a reproductive health and life skill-based intervention on MILs in tribal Rajasthan, India, using a mixed-methods, single-group cluster pilot study.

**Methods:**

We tested a light-touch four-session intervention delivered over 4 months to MILs of newly married women that covered MILs’ health, conception, and communication with DILs and sons and addressed modern healthcare misconceptions, while challenging son preference and fertility norms. This MIL intervention was complemented by a broader 16-session curriculum for newly married women. In July 2023 and January 2024, 42 MILs participated in baseline and endline surveys. We conducted in-depth interviews with 13 MILs and 6 NGO staff members for qualitative insights and to understand barriers and facilitators to the intervention. We also analyzed the data using thematic analysis.

**Results:**

Our results demonstrated the acceptability and feasibility of the intervention (80% attendance, 97.6% satisfaction, 97.6% retention rate). The qualitative findings revealed the impact of the intervention across multiple domains: improved reproductive health knowledge, better gender attitudes and attitudes towards modern healthcare practices, and positive changes in intergenerational relationships. Key implementation barriers included time constraints due to agricultural/household work, while family support and flexible scheduling facilitated participation.

**Conclusion:**

Our findings challenge conventional approaches targeting young women, suggesting the value of including influential family members, such as mothers-in-law, in reproductive health interventions, particularly in societies with extended family structures.

**ClinicalTrials.gov:**

The study is registered at ClinicalTrials.gov (NCT06320964). Registered retrospectively on 13 March 2024, https://clinicaltrials.gov/study/NCT06320964 IRB Approval number: 22 -37,173

## Paper context

- **Main findings:** The four-session light-touch intervention delivered over 4 months was acceptable and feasible to implement, with mothers-in-law demonstrating increased understanding of birth spacing, modern contraception, improved relationship dynamics with their daughters-in-law, and shifted preferences around gender attitudes and having male grandchildren.
- **Added knowledge:** Our findings demonstrate that even a light-touch, targeted intervention can successfully improve intergenerational relationships while addressing reproductive health goals.
- **Global health impact for policy and action:** This study provides evidence supporting the integration of mother-in-law-focused components into existing reproductive and maternal health programs in South Asia, recognizing mothers-in-law as crucial stakeholders in patriarchal-patrilocal family structures.

## Background

Newly married young women in South Asia face significant challenges transitioning into married life, particularly in patriarchal settings [1,2]. Limited opportunities for education and financial independence make them heavily reliant on their husbands and in-laws, especially in patrilocal systems where brides move in with their husbands’ families [3], subjecting young women to the authority of in-laws, creating a complex dynamic that influences their contraceptive behavior and freedom of movement [4].

Mothers-in-law (MILs) often exert more influence on newly married women than their husbands do, especially during the initial years of marriage. Global research consistently shows that MILs shape daughters-in-law’s (DILs) lives, including family planning decisions, resource control, and adherence to gender norms [5–8]. In South Asia, MILs’ presence and attitudes significantly impact DILs’ reproductive health autonomy, with co-residence linked to greater use of modern limiting methods among Indian DILs [4,9].

Many MILs expect DILs to have children immediately or soon after marriage, often preferring male grandchildren due to cultural norms around inheritance and perceived economic benefits [10–12]. This influence extends to limiting DILs’ freedom of movement, peer networks, and social interactions, making it difficult for them to access family planning resources [4]. Additionally, the desire for male grandchildren exerts pressure on DILs to bear sons, perpetuating gender biases within the family [13]. While some MILs may be open to delayed childbirth due to health concerns, social pressures often override these considerations [14].

The relationship between MILs and DILs is crucial in traditional patrilocal-patrilineal family structures in India and elsewhere, where such structures are common [15]. Newly married women often experience ‘double powerlessness’ [16,17]––subordinate to men within the household and to elder women [18–20]. While conflicts between MILs and DILs are common [21–23], research suggests positive MIL-DIL relationships can improve newly married women’s spousal relationships and mental health [24,25].

Despite global calls for MIL involvement in family planning programs, few interventions have targeted MILs of newly married women [9,26,27]. A handful of studies have examined interventions with MILs of newly married women [4,28], with one study focusing on domestic violence prevention through improving MILs’ gender-equitable attitudes [29].

Recognizing MILs’ crucial role in young couples’ reproductive decisions and the dearth of interventions in this setting, our study designed a light-touch intervention for MILs. In this paper, we report on (i) the design of MIL sessions using a community-engaged process, (ii) the feasibility, acceptability, and qualitative impact of the TARANG (Transforming Actions for Reaching and Nurturing Gender Equity and Empowerment) intervention for MILs, while also identifying engagement barriers and facilitators for future interventions.

## Methods

### Study setting

Our study engaged MILs of newly married women in the Kumbhalgarh block of the Rajsamand district in Rajasthan, India, from July 2023 to January 2024, with the intervention spanning 6 months. The block was chosen based on our implementing NGO partner’s operational scope and cultural familiarity with the region. Rajsamand district faces significant family planning challenges with only 50% of the women using any modern contraceptive method, lower than Rajasthan’s state average [30]. Further, among young women in the district who use contraception, female sterilization is the dominant method, indicating limited adoption of reversible short and long-acting contraceptive methods such as condoms, intrauterine devices, and oral contraceptive pills [30].

### Study design

This convergent mixed methods study consisted of a one-group design with pre- and post-intervention data collection to assess intervention acceptability, feasibility, and perceived impact from the perspective of MILs who participated in the light-touch component of the intervention.

### Sample size, participants, and recruitment

The sample size for this pilot study was determined based on feasibility, considering its exploratory nature. As this was a feasibility and acceptability study, formal sample size calculations were not conducted, which aligns with established methodological guidelines for mixed-methods research in pilot and feasibility studies [31,32].

The study enrolled MILs whose DILs met the following criteria: (1) newly married (within the past year), (2) aged 18–25, (3) residing in their marital household for at least 6 months, and (4) co-residing with their MIL. This sampling approach enabled examination of intergenerational dynamics in households where family planning decisions were likely to be actively negotiated. Of 45 approached households, 43 MILs were enrolled at the baseline, with two excluded due to migration [33]. At the time of recruitment, trained research assistants approached MILs who explained the study purpose and obtained verbal informed consent. MILs were explained about the TARANG intervention as a community program focusing on family health and relationships. They were invited to participate in four structured sessions facilitated by female NGO moderators. The intervention was presented as an opportunity to strengthen family relationships and improve health outcomes, with each session addressing specific themes mentioned under the TARANG intervention write-up. The intervention’s underlying aims included promoting gender-equitable attitudes, supporting delayed childbirth, equal valuing of daughters and sons, and enhancing DIL autonomy, but these were approached through the lens of family harmony and wellbeing rather than explicitly challenging traditional authority structures. This light-touch approach was strategically designed to minimize resistance while creating a supportive environment for newly married women in the household. No monetary incentives were given to participants; engagement was encouraged by emphasizing potential benefits to family health and relationships.

### Community-engaged approach for the design of the TARANG intervention for MILs

The TARANG intervention aims to address contraceptive uptake and prevent unintended pregnancy among newly married young women. The intervention covered themes on Empowerment, Norms, and Sexual Reproductive Health, aligning with the broader program for newly married women [34].

The intervention content was developed from February to May 2023 through collaboration between a local NGO in Udaipur, content specialists in Jodhpur, and the research team. The development process included two rounds of iterative testing of the content with 15 MILs in rural and tribal Southern Rajasthan using a community-engaged approach to ensure the content was understandable and culturally appropriate. During these testing sessions, the team assessed four key parameters: session duration required to deliver the session, participant response, toolkit usability, and participant engagement. Feedback-informed refinements enhance the curriculum’s relevance and linguistic suitability for the target audience before proceeding to the feasibility study reported here (July 2023−January 2024). Grounded in a rights-based framework and leveraging adult learning principles, the intervention content was developed in local languages (Hindi and Mewari), incorporating interactive activities and audiovisual aids. A toolkit with materials suitable for older neo-literate populations was created for the patriarchal settings.

### The TARANG intervention for MILs

**Figure 1.**
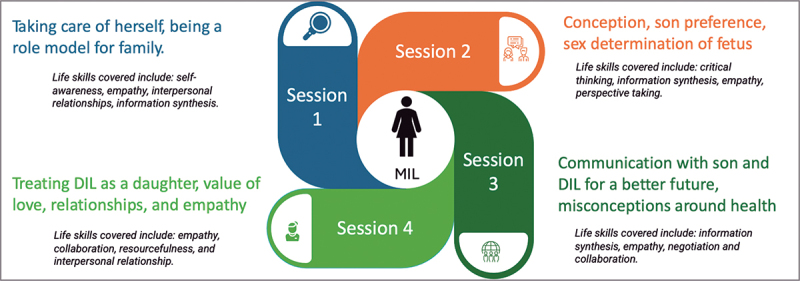
Intervention sessions for MILs.

Female NGO moderators conducted four MIL sessions in each of the four chosen villages in Rajsamand district, Rajasthan. The light-touch intervention addressed nine life skills, including critical thinking and empathy (Figure 1), covering MILs’ health, conception, communication with DILs and sons, and modern healthcare misconceptions. Sessions aimed to challenge son preference and fertility norms while creating a supportive environment for young married women. These sessions complemented the primary 16-session curriculum for newly married women that focused on reproductive health, agency, and decision-making. More details of intervention for newly married women are covered elsewhere [1,34].

The TARANG intervention aimed to promote gender-equitable attitudes by empowering MILs to support family planning, delay early childbirth, and value daughters and sons equally. This approach aimed to enhance family harmony and reduce pressure on DILs to fulfill gendered expectations that impact their health and autonomy [4,35]. However, interventions must consider potential resistance from MILs whose traditional authority may feel challenged, particularly in patrilocal systems. Given the potential for acceptance and resistance to changing traditional roles, particularly in patrilocal systems, evaluating MILs’ responses to the intervention was crucial for understanding implementation facilitators and barriers before rolling out a larger study.

## Data collection procedures

### Quantitative data

Before data collection, we conducted a three-day training session for female enumerators, covering interview techniques, ethical considerations, and survey administration using tablets. We pre-tested the survey instruments with five MILs from a non-study village to refine question wording and response options. Data were collected through baseline and endline surveys with MILs, with the primary outcomes listed in Table 1. Trained female enumerators conducted 45-min computer-assisted personal interviews in participants’ homes or preferred private locations. Moderators also tracked session attendance and reasons for absence using a mobile application throughout the intervention.

**Table 1.** Demographics of MILs in our sample at baseline (*n* = 43).

Demographics	%/mean (SD)	n
Marital Status		
Married	69.7	30
Widowed	30.3	13
Age	49.5 (±10.0)	43
Education		
No education	83.7	36
Any elementary education (1–5)	16.3	7
Caste		
Schedule caste (SC)	14.6	6
Schedule tribe (ST)	63.4	26
Other backward class (OBC)	12.2	5
General category (GC)	7.3	3
Work		
Never gainfully employed	55.8	24
Gainfully employed but not currently	25.5	11
Currently gainfully employed	18.6	8
Number of friends/relatives MIL has in the village	73.8	31
Number of children MIL wants their son and DIL to have		
Don’t know	53.5	23
Up to two	47.6	8
Three or more	27.9	12
Ideal time to first childbirth for their son and DIL		
Don’t know	39.5	17
<1 year	30.3	13
2 years	16.3	7
3 years	13.9	6
Number of grandsons preferred		
More grandsons	67.4	29
More granddaughters	18.6	8
Only granddaughters	4.7	2
No preference	9.3	4
Belief that sex determination is God’s will	95.2	41
Knowledge that sex determination is illegal in India before childbirth in India	55.8	24

### Qualitative data

Post-intervention, using maximum variation sampling, in-depth interviews were conducted with 13 MILs and six NGO staff in October/November 2023. Interviews were conducted by trained qualitative researchers (LG and UC) in Mewari at participants’ homes after obtaining verbal consent, using a guide (Supplementary File: Appendix 1) that explored intervention acceptability, benefits, barriers, and facilitators. On average, interviews lasted about 30 min. Audio recordings were transcribed verbatim in Mewari and then translated into English by bilingual research assistants. To ensure accuracy, a senior researcher (PS) verified 20% of translations against the original recordings to ensure accuracy.

### Quantitative analysis

We analyzed all the quantitative data as means or proportions using Stata 15.1. [45] The primary outcomes were: (a) feasibility: MILs’ attendance greater than at least 50% of the sessions; (b) acceptability: proportion of MILs satisfied with the intervention; (c) usefulness: proportion of MILs that found the TARANG intervention useful/somewhat useful. Before the analysis, we implemented data cleaning procedures, including range checks, consistency checks, and addressing missing values.

### Qualitative thematic analysis

We employed Braun and Clarke’s (2006) approach to thematic analysis [36]. First, three researchers (JV, TP, RR) independently familiarized themselves with the data by reading all transcripts multiple times and recording initial impressions. Second, we developed a preliminary codebook based on the interview guide, incorporating additional codes from the initial transcript review. They were refined through inductive coding. Double-coding was completed for 10% of transcripts and was used to reconcile discrepancies. The codebook was refined based on this process, and the remaining transcripts were coded by a single researcher (LG) using Dedoose version 9.0 [37]. Themes were developed through thematic analysis with supporting quotes [36]. This step was followed by review and editing by co-authors (LG, DD, UC, NDS). This included members of the implementation team in India, ensuring the interpretations accurately reflected the nuances and complexities of the local culture. We enhanced trustworthiness through triangulation of data sources (MILs and NGO staff), analyst triangulation, and reflexive discussions among team members about positionality and potential biases.

#### Data integration

Following a convergent mixed methods design (Creswell & Plano Clark, 2018), we collected quantitative and qualitative data separately but analyzed them separately [38,39]. We present the quantitative and qualitative findings separately in the results section and then draw on both to interpret the findings in the discussion section. Integration occurred primarily at the interpretation phase, where we examined convergence, complementarity, and divergence between the two data sources. This approach allowed us to develop a more comprehensive understanding of intervention acceptability and impact than was possible with either method alone.

## Results

Most MILs (69.7%) were still married, while 30.3% were widowed. The average age of MILs in was 49.5 years. Most (83.7%) had no formal education, with 16.3% having elementary education. The majority belonged to marginalized castes (ST: 63.4%, SC: 14.6%, OBC: 12.2%), with a few from the general category (7.3%). Over half (55.8%) had never worked for money, while 25.5% had past employment. Most (73.8%) reported having local social connections.

About 40% were unsure about the ideal timing for the first pregnancy for their DILs, though 30.3% preferred it within the first year of marriage. Regarding fertility preferences, 53.5% were uncertain about desired grandchildren numbers, while among others, 47.6% preferred two grandchildren and 27.9% preferred three or more. Most MILs (67.4%) preferred grandsons over granddaughters (18.6%), with 9.3% expressing no preference. While 95.2% attributed sex determination to divine will, only 55.8% knew that sex determination before childbirth was illegal in India.

### Quantitative results

The table below presents the primary outcomes and perceived impact outcomes for MIL intervention from endline surveys. The results are presented based on the structure provided in Table 2.

**Table 2.** Feasibility, acceptability, and secondary outcomes for MIL intervention (*n* = 42).

Outcomes	Indicators	% (n)
Feasibility	Mean number of sessions attended	2.8
	Attendance greater than 50% of the sessions*	80.9% (34)
Acceptability	Satisfied with TARANG intervention*	97.6% (41)
	Liked the sessions of TARANG intervention	95% (40)
	Sessions should be held in their village	97.6% (41)
Usefulness	Found the intervention useful*	100% (42)
	Recommend to a friend	100% (42)
	High-level of connection between participants in the groups	90.4% (38)
Perceived impact	Support towards their DILs after completing the intervention	92.8% (39)
	Information from intervention applicable to their lives	85.7% (36)
Retention rate	Recruited at baseline retained until end of the study	97.6% (42)

*Primary outcomes.

MILs’ attendance data showed 80.9% attended more than half the sessions (mean attendance: 2.8 of the total 4) with a 97.6% retention rate. Among participants, 97.6% reported satisfaction, 95% liked the sessions, and 97% supported village-level implementation. All participants (100%) reported the content was valuable and would recommend it to others, with 90% reporting a connection with other group members. Post-intervention, 92.8% reported feeling more supportive toward their DILs, and 86% indicated applying learnings in daily life.

### Qualitative themes

#### Theme 1: acceptability of the TARANG intervention among MILs

Most MILs responded positively to the content of the intervention sessions, expressing appreciation and enjoyment.

*‘We like listening to stories of Kamala (a character in the intervention session) in the meeting, seeing pictures, learn about eating fruits, eating green and yellow vegetables.’ MIL 4, Age 34, Caste: OBC, Education: Illiterate*

Some MILs suggested potential additions for future intervention sessions. One MIL also proposed topics for DILs and their sons, as noted below:

Any livelihood-related information and things that they need to understand should be conveyed to newly married couples so they can be financially empowered too. Any useful information should be provided. Any good information should be available for those who don’t know. MIL 1, Age 50, Caste: SC, Education: Illiterate

The NGO staff and moderators corroborated the acceptability and value of the intervention for MILs.

#### Theme 2: perceived impact on MIL

##### Increased understanding of delayed timing of first childbirth and healthy spacing intervals

Most MILs reported understanding the benefits of birth spacing, emphasizing three-year intervals between children for maternal and child wellbeing. They noted that delayed first childbirth could strengthen son–DIL relationships and demonstrated knowledge of various contraceptive options.

After having one child, there should be a gap of at least three years; when one child grows up, the care of the other can be managed well. Continuous childbirth makes both the children and the mother unhappy. Nowadays, our children understand everything; they can take pills, use condoms, and get injections. They can also use copper-T and have it removed. MIL 5, Age 43, Caste: ST, Education: Illiterate

NGO moderators noted MILs’ receptivity to family planning information and retention of key concepts.*‘Mother-in-law has also accepted that having children should not be rushed, and that having two or three children is sufficient. There should be a gap between the children; the first child should become intelligent, and grow up, and then the second one should be considered.’ NGO Staff 1.*

##### Shift in cultural and gender attitudes and social norms

Many MILs recalled sessions challenging gender norms, particularly through the ‘seed’ activity demonstrating sex determination concepts. They reported shifts in traditional beliefs about son preference and women’s role in determining a child’s sex.

*‘We were taught (in the program) not to differentiate between sons and daughters. They used an interesting game- seed activity, which made it interesting for me …’ MIL 2, Age: 47, Caste: SC, Education: Illiterate*

*‘It’s not in one’s hands. Both should be responsibly taken care of. It’s not solely in the woman’s hands whether it’s a boy or a girl.’ MIL 9, Age: 52, Caste: SC, Education: Illiterate*

Furthermore, some MILs mentioned shifting away from traditional practices like home births, toward modern healthcare practices such as hospital deliveries, using contraceptives for family planning and spacing children, and prioritizing pregnancy care for their DILs.

Everything was good. When two different seeds (referring to a game using seeds on sex determination) come together, it’s a boy, and when similar seeds come together, it’s a girl. I learned about having a gap between children, I’ll tell my daughter-in-law about the gap between children and talk to her about it. If needed, we’ll take her (DIL) to the hospital. In my time, we didn’t go to the hospital; deliveries happened at home. While doing household work, we had five children. MIL 5, Age 43, Caste: ST, Education: Illiterate

##### Increased knowledge of health and well-being among MILs

While TARANG primarily focused on family planning and gender norms, MILs learned about nutrition, health, pregnancy care for DILs, and hospital services. They valued this learning for its direct impact on their family caregiving roles and household management.

All discussions were good, especially those about cleanliness, food and drink, going to the hospital at the time of the delivery, explaining about children, using contraceptives and talking about seeds activity (referring to a game that taught them sex determination of a child). The information shared is good – we learn about how to take care of ourselves, our nutrition, our body. Information was given in the meeting that family members should sit together, decide what to do or not, listen, and understand each other. MIL 8, Age 55, Caste: ST, Education: Illiterate

##### Changes in relationship dynamics of MIL-DIL and other household members

MILs reported that TARANG sessions enhanced their understanding of family relationships, particularly with DILs. They emphasized treating DILs with respect and as equal family members, supporting their empowerment through education.

When the women gather, they discuss things, and it lightens the mood. In the meeting, we get to hear good things. We should treat daughter-in-law with respect. She is also like a daughter, and now we are her parents. The daughter-in-law and daughter should be treated equally. We learned that there should be a deep relationship between mother-in-law and daughter-in-law. Everyone should live together, and there should be no bitterness. A family is very important. If positive story about my family spreads, everyone will accept that this family lives together in harmony. MIL 3, Age 38, Caste: OBC, Education: Illiterate

MILs described that TARANG had improved their two-way family communication and joint decision-making: *‘My daughter-in-law and I talk. We both learn things in the meetings and explain them to each other. I also started to communicate with all family members, respecting their opinions, and making decisions together after going to the meetings.’ MIL 5, Age 43, Caste: ST, Education: Illiterate*

They actively encouraged DIL participation in sessions, demonstrating support for their learning: *‘I tell my daughters-in-law that they should attend the meetings. I can manage the household chores, and there’s no objection to learning good things by sitting with knowledgeable people.’ - MIL 12, Age: 48, Caste: ST, Education: Illiterate*

The NGO moderators also confirmed the transformation of MILs, providing important triangulation of the self-reported data from MILs:

Radha (name changed) didn’t attend the first session, then her mother-in-law encouraged her to participate for an hour. There’s a mother-in-law who suggests taking her daughter-in-law to the meeting, saying she will learn something. NGO Moderator 1

#### Theme 3. Barriers and facilitators

##### Barriers to intervention implementation and attendance

Many MILs faced attendance challenges due to busy daily routines, including household and agricultural work: *‘No one stops me, but it’s hard to find time.’ - MIL 8, Age: 55, Caste: ST, Education: Illiterate*

Feeding cows and goats, spreading manure, making tea, drinking, going to the fields. If there’s fodder to cut, then I go there. After coming back from the field, I bathe and wash clothes. Household chores keep me busy all day. In the evening, cook dinner, eat, and then go to sleep around 10–11 PM. MIL 2, Age: 47, Caste: SC, Education: Illiterate

NGO staff and moderators noted sporadic attendance due to scheduling challenges, with festivals, weddings, and work commitments affecting participation.

MIL is working in NREGS (National Rural Employment Guarantee Scheme, a rural employment scheme that provides minimum work for 100 days in exchange of wages), it’s challenging to have sessions as per our schedule. It’s not possible to schedule all sessions on Thursdays because Thursdays are a holiday for NREGS. The third challenge is that going to the field in the morning and evening and then finding time afterwards is not possible. – NGO staff

Some MILs believed the sessions were more relevant for DILs:

MIL says, why don’t you take meetings of newly married women (referring to their DIL), we don’t need it, explain to them. If they are pregnant, at least they will know how to take care of their body. This information will be available through such programs. NGO Moderator 2

##### Facilitators that created an enabling environment to continue attending intervention sessions

MILs reported family support or at least no opposition to their session attendance, often managing household responsibilities around meeting times:

No, there is no problem. After finishing all the household work, I attend the meeting, so there is no trouble for family members. MIL 5, Age: 43, Caste: ST, Education: Illiterate

NGO moderators emphasized the importance of building community trust:

Initially, no one used to come to the meetings. Then I sat with them, created a friendly behavior, and then went to different homes. trust must be built with the father-in-law, as he is the head of the family. So, first, the father-in-law and mother-in-law need to be informed about the program, and its benefits need to be explained. Initially, they didn’t like it either. Interest was generated in them through group activities like dancing, laughter, and jokes that attending the meeting would be beneficial. NGO Moderator 2

Moderators also reported that flexible scheduling and transportation support also facilitated attendance:

We have to adjust the session time accordingly. With homes being far away, it requires bringing them in a vehicle and dropping them back. NGO moderator 2

#### Theme 4. Program improvements

MILs suggested scheduling sessions around work commitments, preferring afternoon or evening timings: *‘Meetings should be scheduled during free time so that everyone can attend … after 3 to 5 in the afternoon [is a good time].’ – MIL 01, Age: 50, Caste: SC, Education: Illiterate*

MILs’ views on session length varied, with some noting family concerns about longer meetings: ‘*One and a half hours are sufficient. If the time is more, family members will question why so much time is being spent.’ MIL 4, Age: 34, Caste: OBC, Education: Illiterate*

Several MILs requested additional content on government schemes, livelihoods, and health, preferring visual and interactive learning methods over written activities, which moderators agreed were more effective for neo-literate participants.

## Discussion

In this pilot study from tribal Rajasthan, our mixed methods evaluation found the light-touch TARANG intervention for mothers-in-law (MIL) acceptable and feasible, based on participation rates, logistical factors, and participant feedback. The intervention showed promise in improving knowledge, MIL-DIL relationships, and challenging gender-related social norms. TARANG demonstrates potential for enhancing MILs’ roles in family planning interventions.

We found varied fertility preferences among MILs, with most unsure about the number of grandchildren, with preferences ranging from small to large families. There was also variability in opinions on the timing of the first childbirth, with some advocating for early childbearing and many expressing uncertain opinions. Limited awareness about illegal sex determination persisted, aligning with previous studies [9,13]. These findings highlight the need for MIL-focused interventions to promote informed reproductive health decisions [23].

Quantitative findings strongly supported the intervention: 80% of MILs attended at least 50% of sessions, with 97.6% reporting high satisfaction. All participants found the content useful. Importantly, by the end of the intervention, 92.8% of the participants reported feeling supportive towards their DILs. These results underscore MILs’ crucial role in family planning decisions among young married couples in South Asia, including India [4,5,11].

Qualitative analysis reinforced the quantitative results. The intervention enhanced MILs’ understanding of family planning and child spacing, with MILs recognizing the benefits of delayed first childbirth and pregnancy spacing. MILs demonstrated shifts in traditional beliefs about son preference, acknowledging that fetal sex determination was beyond women’s control. They also showed increased acceptance of modern healthcare practices, including hospital deliveries and contraceptive methods, indicating positive changes in attitudes toward maternal and child health.

The intervention showed promise in improving MIL-DIL relationships, with MILs emphasizing respect, equality, and family harmony. They actively encouraged DILs’ participation in sessions and demonstrated genuine interest in their well-being. This engagement is aligned with our goal of increasing DILs’ participation and empowering MILs as advocates for family planning. The downstream effect observed in quantitative and qualitative results mirrors findings from a similar pilot intervention in Nepal involving MILs of newly married couples, highlighting MILs’ potential as agents of change [28]. The complementary analysis of DIL perspectives in our forthcoming paper (currently under review) will provide an important counterpoint to assess the extent of convergence between MIL and DIL accounts [1].

Despite positive outcomes, MILs faced attendance challenges due to household, agricultural, and employment commitments. Family support emerged as a crucial facilitator, with members providing approval and practical assistance with household responsibilities. The implementing NGO addressed barriers through community trust-building, flexible scheduling, and logistical support, which proved essential for sustained participation.

Based on participant feedback, NGO staff insights, and data analysis, we refined the intervention while maintaining its light-touch four-session structure for the main randomized controlled trial. Modifications included additional MIL-focused health content (menopause, care-seeking), flexible session scheduling over 5–6 months, and enhanced visual and narrative-based learning materials to improve engagement.

Several limitations deserve consideration. The modest sample size, although sufficient for testing study procedures and refining the intervention for a future trial, restricts the generalizability of our findings. The lack of accurate eligible household listings prevented the calculation of recruitment rates. Finally, it is worth noting that we were not able to quantitatively measure the intervention’s impact on MILs’ knowledge, attitudes, and behaviors. Any attempt to assess quantitative impact would have been primarily descriptive.

Despite these limitations, our study offers several strengths. First, our comprehensive mixed methods approach, integrating quantitative surveys, in-depth interviews, and monitoring data, provided a nuanced understanding of the intervention’s feasibility and acceptability. Second, our intervention development process ensured a community-engaged approach, enhancing the cultural relevance and acceptance of the intervention. Third, implementation in representative villages of rural/tribal Southern Rajasthan provides valuable groundwork for our forthcoming cluster randomized controlled trial and insights for future interventions aimed at MILs in similar contexts.

The detailed documentation of intervention development and modules contributes to implementation science, facilitating adaptation in diverse settings. While the intervention’s goals of promoting gender-equitable family norms and improving reproductive health decision-making are broadly relevant, successful implementation may require careful tailoring to local cultural dynamics and family structures. Such adaptations will be crucial for ensuring meaningful engagement across diverse populations. Additionally, practical elements like session timing, duration, and frequency may need adaptation to address regional accessibility and logistical variations. This analysis emphasizes balancing core intervention objectives with local adaptations to enhance cultural sensitivity and program sustainability. Understanding community-specific barriers and facilitators is crucial for meaningful implementation across diverse settings.

## Conclusion

In the context of South Asian patriarchal-patrilocal joint families, MILs significantly influence gender norms, often promoting early fertility and son preference among newlywed women. Our mixed methods pilot study demonstrated that the intervention was acceptable and feasible to implement with MILs. The intervention showed promise in enhancing knowledge and attitudes, improving MIL-DIL relationship dynamics, and challenging traditional norms around male child preference and early childbearing. These findings establish a foundation for future interventions aimed at engaging MILs as agents of change in the lives of young married women, fostering supportive environments for improved reproductive health outcomes and gender equity. The success of this approach suggests the value of integrating MIL-focused components into existing reproductive and maternal health programs in similar extended family contexts in South Asia and beyond.

## Supplementary Material

Appendix 1.docx

## Data Availability

The data that support the findings of this study are openly available in the Harvard Dataverse at https://doi.org/10.7910/DVN/U821RR

## Appendix 1: Interview Guide for the Pilot Evaluation Qualitative Study – Beneficiaries/Participants

### Icebreakers

1. Can you tell me a little bit about your everyday life or can you describe a typical day in your life? What are your daily routines, activities, and responsibilities?
2. Can you tell me a little bit about your family – Who are the members of your household? And what are their roles and relationships within the family? (can include the second part or even skip).

As you know, our TARANG program is for young married couples and mothers-in-law in this village. I want to learn about your experience of attending this program and get your feedback on it and how you think it has helped you.

3. Can you tell us about involvement with the TARANG program?

- What are your thoughts on the program (probe for how MIL support or engage with the program in case of husband or newly married woman)?
- What are some things that you like/liked about it?
- What are some things that you did not/don’t like?
- How does it feel to attend the sessions?

**a. Add probes**: Do specific emotions come to mind, such as happiness or nervousness?

- What sessions have really been most meaningful for you, and what makes them stand out?
- How would you like to see the community and other family members involved in this program? (Probe for how in-laws should or should not be included, how peer support could be useful or how other community members could be engaged? Are there particular roles or responsibilities you believe other community members could take on to support this initiative?)

4. Did you face any barriers or facilitators for you attending the sessions?

a) What made it possible for you to attend? (This is to get at facilitators and barriers to attendance)

- e.g., support of in-law (or lack thereof), support of husband (or lack thereof),
- e.g., holidays/free time/leisure/extra time
  a. What has made it easier for you to attend?
  b. What made it harder for you to attend? OR what can be done to make it easier for you to attend?
  c. Were you able to overcome those barriers? If you managed to overcome any of these barriers, could you share how you did so and whether these strategies could be helpful for others?
  How could we make it easier for people to attend?
    (1) Specifically thinking about the following things, what can be done to make the program more relevant or improve the program?
      a. Length of the sessions
      b. Timing of the sessions
      c. Content of the sessions
      d. Composition of the group
      e. Follow-up and continuity
      Local context and cultural sensitivity
      Feedback mechanisms
      Moderators’ delivery
      Moderators’ approach to the sessions?
      What was missing in our sessions that you think we should have covered?
    What kind of benefits have you seen because of this program?

a) What kind of personal benefits did you have from attending this TARANG program? (probe one by one).

- Knowledge of family planning/fertility awareness.
- Attitudes towards family planning/fertility awareness.
- Practices of family planning.
- Time to first birth.
- Spacing between children.
- Changed Attitudes
- Ability to negotiate.
- Ability to decide for yourself.
- Enhanced Practices

b) Let’s talk about benefits as a family.

- Relationship
- Communication
- Trust
- Support to attend sessions/other events/freedom of movement.
- Decision-making
  a. What, if any, did you observe as costs/downsides (expected and unexpected) to attending these sessions? What is it?
  b. Personal costs (such as time/not able to do housework/attend office)
  c. Relational costs (such as conflict increase, less support of in-laws/husband/family)

7. Thinking of people who are resistant to family planning in your family/household, how can their attitudes be changed to be more receptive to family planning and family planning methods? Have you tried engaging in open and respectful discussions with these family members about family planning? If so, what approaches have you found effective in fostering understanding and acceptance?

**a. Probe**: For example, who in your family is resistant to you adopting family planning methods and then think how can their attitudes be changed?

8. Are there any other ideas or comments you have for the TARANG program?

a. Any topics you would like more information about? Less information about?
b. If you could suggest any improvement or change to the TARANG program, what would it be, and how do you think it would enhance the program’s effectiveness or relevance to the community?

(1) To what extent will you recommend TARANG to others in your community?
(2) Could you share specific ways in which the TARANG program has directly impacted your life and your family’s well-being?
(3) Can you describe any strong networks or support systems that have developed among you and your peers who are attending TARANG sessions? How have these relationships benefited you in the context of family planning?

## References

[1] Gopalakrishnan L, Patil S, Das D, et al. Feasibility and acceptability of a life skills and reproductive health empowerment intervention for young newly married women in Rajasthan, India: a pre-post convergent mixed methods pilot study. [internet]. Research Square; 2024 [cited 2024 Dec 17]. Available from: https://www.researchsquare.com/article/rs-4255712/v1 10.1186/s40814-025-01720-7 PMC12619424 41241746

[2] Lawrence PG, Hensly C. Gender-based policies and the role of patriarchal norms: evidence from northern India. Fem Econ. 2023 Apr 3;29:252–11. doi: 10.1080/13545701.2023.2168025

[3] Kanji S, Carmichael F, Darko C, et al. The impact of early marriage on the life satisfaction, education and subjective health of young women in India: a longitudinal analysis. J Dev Stud. 2024 May 3;60:705–723. doi: 10.1080/00220388.2023.2284678

[4] Anukriti S, Herrera-Almanza C, Pathak PK, et al. Curse of the mummy-ji: the influence of mothers-in-law on women in India†. Am J Agric Econ. 2020;102:1328–1351.

[5] Char A, Saavala M, Kulmala T. Influence of mothers-in-law on young couples’ family planning decisions in rural India. Reprod Health Matters. 2010 May;18:154–162. doi: 10.1016/S0968-8080(10)35497-8 20541094

[6] Dixit A, Bhan N, Benmarhnia T, et al. The association between early in marriage fertility pressure from in-laws’ and family planning behaviors, among married adolescent girls in Bihar and Uttar Pradesh, India. Reprod Health. 2021 Mar 9;18:60. doi: 10.1186/s12978-021-01116-9 33750403 PMC7941884

[7] Gopalakrishnan L, Bertozzi S, Rabe-Hesketh S, et al. Role of marriage, motherhood, son preference on adolescent girls’ and young women’s empowerment: evidence from a panel study in India. PLOS ONE. 2023 Sep 28;18:e0292084. doi: 10.1371/journal.pone.0292084 37769003 PMC10538655

[8] Ragavan M, Iyengar K. Violence perpetrated by mothers-in-law in northern India: perceived frequency, acceptability, and options for survivors. J Interpers Violence. 2020 Sep 1;35:3308–3330. doi: 10.1177/0886260517708759 29294754

[9] Pradhan MR, Mondal S. Examining the influence of mother-in-law on family planning use in South Asia: insights from Bangladesh, India, Nepal, and Pakistan. BMC Womens Health. 2023 Aug 9;23:418. doi: 10.1186/s12905-023-02587-7 37553598 PMC10410985

[10] Arnold F, Choe MK, Roy TK. Son preference, the family-building process and child mortality in India. Popul Stud. 1998 Nov 1;52:301–315. doi: 10.1080/0032472031000150486

[11] Dixit A, Ghule M, Rao N, et al. Qualitative examination of the role and influence of mothers-in-law on young married couples’ family planning in rural Maharashtra, India. Glob Health Sci Pract. 2022 Oct 31;10:e2200050. doi: 10.9745/GHSP-D-22-00050 36316150 PMC9622279

[12] Jayachandran S. The roots of gender inequality in developing countries. Annu Rev Econ. 2015 Aug 2;7:63–88. doi: 10.1146/annurev-economics-080614-115404

[13] Robitaille MC, Chatterjee I. Mothers-in-law and son preference in India [internet]. Rochester (NY); 2014 [cited 2024 Apr 4]. Available from: https://papers.ssrn.com/abstract=2208354

[14] Diamond-Smith N, Plaza N, Puri M, et al. Perceived conflicting desires to delay the first birth: a household-level exploration in Nepal. Int Perspect Sex Reprod Health. 2020 Jul 23;46:125–133. doi: 10.1363/46e9420 32723708 PMC7433350

[15] Allendorf K. Like her own: ideals and experiences of the mother-in-law/daughter-in-law relationship. J Fam Issues. 2006 Dec 1;55:588–600. doi: 10.1111/j.1741-3729.2006.00428.x 27147777 PMC4852487

[16] Das Gupta M. Lifeboat versus corporate ethic: social and demographic implications of stem and joint families. Soc Sci Med. 1999 Jul 1;49:173–184. doi: 10.1016/S0277-9536(99)00096-9 10414827

[17] Zuo J. Rethinking family patriarchy and women’s positions in presocialist China. J Marriage Fam. 2009;71:542–557. doi: 10.1111/j.1741-3737.2009.00618.x

[18] Skinner GW. Family systems and demographic processes. Anthropological Demography [Internet]; 1997 [cited 2021 Nov 14]. Available from: https://press.uchicago.edu/ucp/books/book/chicago/A/bo3617150.html

[19] Das Gupta M. Life course perspectives on women’s autonomy and health outcomes. Am Anthropol. 1995;97:481–491. doi: 10.1525/aa.1995.97.3.02a00070

[20] Das Gupta M. Life course perspectives on women’s autonomy and health outcomes. Health Transit Rev. 1996;6:213–231.

[21] Allendorf K. Women’s agency and the quality of family relationships in India. Popul Res Policy Rev. 2012 Apr 1;31:187–206. doi: 10.1007/s11113-012-9228-7 27147776 PMC4852544

[22] Schensul SL, Brault MA, Prabhughate P, et al. Sexual intimacy and marital relationships in a low-income urban community in India. Cult Health SEx. 2018 Oct 17;20:1087–1101. doi: 10.1080/13691058.2018.1491060 PMC6470050 30328771

[23] Anukriti S, Herrera-Almanza C, Pathak P, et al. Curse of the mummy‐ji: the influence of mothers‐in‐law on women in India†. Am J Agric Econ. 2020 Aug 23;102:1328–1351.

[24] Bryant CM, Conger RD, Meehan JM. The influence of in-laws on change in marital success. J Marriage Fam. 2001;63:614–626. doi: 10.1111/j.1741-3737.2001.00614.x

[25] Gopalakrishnan L, Acharya B, Puri M, et al. A longitudinal study of the role of spousal relationship quality and mother-in-law relationship quality on women’s depression in rural Nepal. ssm - Ment Health. 2023 Dec 1;3:100193. doi: 10.1016/j.ssmmh.2023.100193 PMC13037867 41924583

[26] Behera J, Mehra S, Dhal Samanta S, et al. Planning & designing reproductive health intervention framework for young married couples in India: using systematic review. Indian J Public Health Res Dev. 2019 Jan 1;10:20. doi: 10.5958/0976-5506.2019.01232.4

[27] White D, Dynes M, Rubardt M, et al. The influence of intrafamilial power on maternal health care in Mali: perspectives of women, men and mothers-in-law. Int Perspect Sex Reprod Health. 2013 Jun;39:058–068. doi: 10.1363/3905813 23895882

[28] Mitchell A, Puri MC, Dahal M, et al. Impact of sumadhur intervention on fertility and family planning decision-making norms: a mixed methods study. Reprod Health. 2023 May 25;20:80. doi: 10.1186/s12978-023-01619-7 37231469 PMC10211300

[29] Krishnan S, Subbiah K, Khanum S, et al. An intergenerational women’s empowerment intervention to mitigate domestic violence: results of a pilot study in Bengaluru, India. Violence Against women. 2012 Mar 1;18:346–370. doi: 10.1177/1077801212442628 22531083

[30] International Institute for Population Sciences (IIPS), ICF. National family health survey 5 (NHFS-5): 2019–2021. Mumbai: IIPS; 2022.

[31] Orsmond GI, Cohn ES. The distinctive features of a feasibility study: objectives and guiding questions. OTJR Occup Particip Health. 2015 Jul;35:169–177. doi: 10.1177/1539449215578649 26594739

[32] O’Cathain A, Hoddinott P, Lewin S, et al. Maximising the impact of qualitative research in feasibility studies for randomised controlled trials: guidance for researchers. Pilot Feasibility Stud. 2015;1:32. doi: 10.1186/s40814-015-0026-y 27965810 PMC5154038

[33] National Family Health Survey. (NFHS-5) India 2019–21. India: International Institute for Population Sciences; 2022.

[34] Diamond-Smith N, Gopalakrishnan L, Hannah L, et al. A life skills and reproductive health empowerment intervention for newly married women and their families to reduce unintended pregnancy in India: protocol for the TARANG cluster randomized controlled trial. BMJ Open. 2024;14:e086778. doi: 10.1136/bmjopen-2024-086778 PMC11086273 38688674

[35] Gopalakrishnan L, Diamond-Smith N, Acharya B, et al. The relationship between the gendered norm of eating last and mental health of newly married women in Nepal: a longitudinal study. Matern Child Nutr. 2023 Jul;19:e13508. doi: 10.1111/mcn.13508 36994887 PMC10262909

[36] Braun V, Clarke V. Using thematic analysis in psychology. Qual Res Psychol. 2006 Jan 1;3:77–101. doi: 10.1191/1478088706qp063oa

[37] Dedoose Version 9.0.17. Web application for managing, analyzing, and presenting qualitative and mixed method research data. 2021.

[38] Fetters MD, Curry LA, Creswell JW. Achieving integration in mixed methods designs—principles and practices. Health Serv Res. 2013 Dec;48:2134–2156. doi: 10.1111/1475-6773.12117 24279835 PMC4097839

[39] Creswell J, Clark V. Designing and conducting mixed methods research. 2nd ed. Thousand Oaks (CA): Sage Publications; 2010.

