# Supplementary material for: Implementation and evaluation of a family planning intervention engaging mothers-in-law of young women in India: a mixed methods pilot study
Source: Glob Health Action. 2025 Sep 8;18(1):2554435. doi: 10.1080/16549716.2025.2554435 (PMC12418791; doi:10.1080/16549716.2025.2554435)
Supplement: Appendix 1.docx [file ZGHA_A_2554435_SM0706.docx]

**Appendix 1: Interview Guide for the Pilot Evaluation Qualitative Study – Beneficiaries/ Participants**

**Icebreakers**

1. Can you tell me a little bit about your everyday life. or can you describe a typical day in your life? What are your daily routines, activities, and responsibilities?
2. Can you tell me a little bit about your family – Who are the members of your household? and what are their roles and relationships within the family? (can include the second part or even skip)

As you know, our TARANG program for young married couples and mothers-in-law in this village. I want to learn about your experience of attending this program and get your feedback on it and how you think it has helped you.

1. Can you tell us about involvement with TARANG program?
2. What are your thoughts on the program (probe for how MIL support or engage with the program in case of husband or newly married woman)?
3. What are some things that you like/liked about it?
4. What are some things that you did not /don’t like?
5. How does it feel to attend the sessions?
   1. Add probes: Do specific emotions come to mind, such as happiness or nervousness?
6. What sessions have really been most meaningful for you, and what makes them stand out?
7. How would you like to see the community and other family members involved in this program? (Probe for how in-laws should or should not be included, how peer support could be useful or how other community members could be engaged? Are there particular roles or responsibilities you believe other community members could take on to support this initiative?)
8. Did you face any barriers or facilitators for you attending the sessions?
9. What made it possible for you attend? (This is to get at facilitators and barriers to attendance)
   - 1. E.g., support of in-law (or lack thereof), support of husband (or lack thereof),
     2. E.g., holidays/ free time/leisure/ extra time
10. What has made it easier for you to attend?
11. What made it harder for you to attend? OR what can be done to make it easier for you to attend?
    1. Were you able to overcome those barriers?. If you managed to overcome any of these barriers, could you share how you did so and whether these strategies could be helpful for others?
    2. How could we make it easier for people to attend?
12. Specifically thinking about the following things, what can be done to make the program more relevant or improve the program?
13. Length of the sessions
14. Timing of the sessions
15. Content of the sessions
16. Composition of the group
17. Follow-up and continuity
18. Local context and cultural sensitivity
19. Feedback mechanisms
20. Moderators’ delivery
21. Moderators’ approach to the sessions?
22. What was missing in our sessions that you think we should have covered?
23. What kind of benefits have you seen because of this program?
24. What kind of personal benefits did you have from attending this TARANG program? (probe one by one).
    - 1. Knowledge of family planning / fertility awareness.
      2. Attitudes towards family planning / fertility awareness.
      3. Practices of family planning.
      4. Time to first birth.
      5. Spacing between children.
      6. Changed Attitudes
      7. Ability to negotiate.
      8. Ability to decide for yourself.
      9. Enhanced Practices
25. Let’s talk about benefits as a family.
    - 1. Relationship
      2. Communication
      3. Trust
      4. Support to attend sessions / other events/ freedom of movement.
      5. Decision-making
26. What, if any, did you observe as costs/downsides (expected and unexpected) to attending these sessions? What is it?
    1. Personal costs (such as time/not able to do housework/ attend office)
    2. Relational costs (such as conflict increase, less support of in-laws/husband/family)
27. Thinking of people who are resistant to family planning in your family/household, how can their attitudes be changed to be more receptive to family planning and family planning methods? Have you tried engaging in open and respectful discussions with these family members about family planning? If so, what approaches have you found effective in fostering understanding and acceptance?
    1. Probe: For example, who in your family is resistant to you adopting family planning methods and then think how can their attitudes be changed?
28. Are there any other ideas or comments you have for the TARANG program?
    1. Any topics you would like more information about? Less information about?
    2. If you could suggest any improvement or change to the TARANG program, what would it be, and how do you think it would enhance the program's effectiveness or relevance to the community?
29. To what extent will you recommend TARANG to others in your community?
30. Could you share specific ways in which the TARANG program has directly impacted your life and your family's well-being?

11. Can you describe any strong networks or support systems that have developed among you and your peers who are attending TARANG sessions? How have these relationships benefited you in the context of family planning?
